# Obsessive-Compulsive Disorder During the COVID-19 Pandemic—A Systematic Review

**DOI:** 10.3389/fpsyt.2022.806872

**Published:** 2022-03-25

**Authors:** Elisabeth S. Linde, Tibor V. Varga, Amy Clotworthy

**Affiliations:** Section of Epidemiology, Department of Public Health, University of Copenhagen, Copenhagen, Denmark

**Keywords:** obsessive-compulsive disorder, OCD, COVID-19, Coronavirus, obsession, pandemic, systematic review, mental health

## Abstract

**Background:**

The COVID-19 pandemic and its associated restrictions may contribute to a deterioration in mental health; individuals with obsessive-compulsive disorder (OCD) may be particularly affected. This systematic review aimed to investigate the effects of the current pandemic on people diagnosed with OCD, and whether pandemics may affect the development of OCD symptoms.

**Methods:**

We conducted a systematic search using NCBI PubMed, SCOPUS, and Google Scholar on February 9, 2021. Research articles related to OCD and COVID-19 or other pandemics were attempted to be identified using pre-defined search terms. Case reports, clinical guidelines, letters, and clinical research articles including ≥100 participants were included; reviews were excluded. The systematic review adheres to PRISMA guidelines and the Newcastle-Ottawa Scale was used to assess the quality of the included clinical research articles.

**Results:**

A total of 79 articles were included in the full-text assessment. Of these, 59 were clinical research articles, two were clinical guidelines, six were case reports, and 12 were letters. The research articles examined OCD symptoms in adult patients with diagnosed OCD, the general population, pregnant women, healthcare workers, students, and young adults, children, and adolescents. Only one study on OCD in previous pandemics was identified.

**Conclusion:**

This systematic review found that people both with and without diagnosed OCD prior to the pandemic generally experienced a worsened landscape of symptoms of OCD during the COVID-19 pandemic. However, the responses are heterogeneous and many factors other than the pandemic seemed to affect the development of OCD symptoms. To prevent the impairment of symptoms and the development of new cases, close monitoring of patients with OCD and education of the general public is essential. Literature is still limited; thus, multinational and cross-cultural, longitudinal studies are warranted to gain further insights on this topic.

## Introduction

Throughout history, pandemics have struck human societies and caused millions of deaths, economic depressions, and even the fall of empires (1). While *epidemic* describes a disease that infects large groups of people within a population or region, the word *pandemic* refers to an epidemic that spreads worldwide and is difficult to contain (2). Pandemics have the ability to shape cultures, politics, religion, health care, and people's mental health for many generations to come (1). Research into pandemics from recent decades also indicates an immediate and long-term negative psychosocial impact on large numbers of individuals (3).

The Coronavirus disease 2019 (COVID-19) pandemic—which was caused by the SARS-CoV-2 virus originating in Wuhan, China, in December 2019—has increased mortality worldwide (4). As of 16 February 2022, there were over 414 million confirmed cases and over 5.8 million deaths worldwide due to infection from the virus and its complications (5). The virus presents with a wide range of symptoms: from milder symptoms like fever, dry cough, and fatigue to severe symptoms like difficulty to breathe, fever, and chest pain (6). About one in six individuals experience complications of COVID-19, some of which are life-threatening (7).

Since the World Health Organization (WHO) declared a pandemic in March 2020 (8), rigorous strategies have been imposed worldwide to limit the spread of SARS-CoV-2. At times, these strategies have included quarantines, physical distancing, and national campaigns on the importance of hand hygiene and wearing protective facemasks (9). It has been reported that fear of the virus and various strategies to limit the virus' spread might have a synergistic effect in exerting a negative impact on the mental health of populations worldwide (10). Quarantines, in particular, may contribute to negative psychological effects (11). According to recent literature, individuals who had been diagnosed with obsessive-compulsive disorder (OCD) prior to the current pandemic may be the group most affected by the pandemic among those with mental disorders (10).

To date, it remains unknown to what extent and through which mechanisms pandemics affect the mental health of people with OCD (12). OCD is a severe anxiety disorder involving uncontrollable obsessions and repetitive compulsions (9). Obsessions are defined as repeated, unwanted thoughts that generate anxiety, whereas compulsions are defined as behaviors subsequent to an obsessive thought (9). OCD is an extremely heterogeneous and idiosyncratic disorder. However, Rajkumar et al. (13) suggest that at least four distinctions can be identified in patients: (a) fear of contamination and cleaning/washing compulsion; (b) obsessive taboo thoughts and checking compulsions; (c) obsessions and compulsions regarding symmetry; and (d) hoarding.

The etiology of OCD is largely unknown but probably consists of a complex combination of both genetic, biological, and environmental factors (14, 15). Evolutionarily, OCD symptoms like contamination fear, handwashing, and hoarding may have developed to protect our ancestors from infectious diseases and from starvation during times of limited resources (13). General risk factors that are known to cause or trigger OCD are stressful life events, comorbid mental-health disorders, a family history of OCD, and/or personality traits like perfectionism, intolerance of uncertainty, and threat overestimation (15–17). High-risk groups include OCD patients in remission/recovery, geriatrics (i.e., people over age 65), pregnant women, children and adolescents, and healthcare professionals (18–20). OCD is associated with reduced quality of life, various comorbid mental disorders and, with severe OCD, an increased risk of suicide attempts (21, 22). The lifetime prevalence of OCD is estimated to be 1.9–2.5% globally (23). Mild symptoms are reported to occur in up to 14–29% of populations, which means that a sizeable proportion of individuals experience symptoms during their lifetime (24).

About 50% of individuals living with OCD worldwide experience symptoms such as a fear of contamination, excessive handwashing, and a fear of dirt (25). Based on current COVID-19 recommendations from WHO, individuals with OCD are encouraged to engage in cleaning habits that were previously considered irrational. Symptoms such as irritability, anxiety, and sadness—which were once restricted to patients with OCD when they came into contact with objects considered contaminated—are now observed in individuals without previous mental disorders (12, 26, 27). This has raised concerns about how to separate rational fears and behaviors exhibited during the COVID-19 pandemic from obsessive fears and compulsions typical of individuals with OCD. Aardema et al. (12) argue that one aspect separating the two groups is the psychological meaning attached to “contamination.” The authors suggest that individuals with contamination fear typically attribute personifications to viruses and germs, which thereby threatens their identity and causes inner corruption (i.e., a threat to the self). The authors explain that OCD is not only characterized by an increased fear of certain threats but also whether these threats target the individual's vulnerable self-theme; i.e., the fear of becoming a certain type of person and/or the areas where the person feels vulnerable and wrong (12, 28).

It has been hypothesized that, during a pandemic, individuals with OCD might believe that their fears of contamination are verified or even encouraged, or they might demonstrate a disproportionate concern about getting infected by the disease (10). During the current pandemic, these phenomena might occur as some of the measures to prevent COVID-19 transmission are similar to behaviors demonstrated by people with OCD, especially those with symptoms like contamination fear and compulsive handwashing (10). Although the emergent crisis of the management of OCD during pandemics is evident, literature is still limited. Thus, an investigation is warranted to learn more about the etiology of OCD and the possible consequences of pandemics on mental health. The goals of this systematic review were to analyze the available evidence in order to gain knowledge about: (1) whether the COVID-19 pandemic has increased the prevalence of OCD symptoms; (2) which specific demographic groups are the most susceptible and which personal characteristics contributed to the worsening of OCD symptoms; and (3) whether there are recommendations on how to improve the management of OCD during the current and future pandemics.

This systematic review of the literature includes both articles that report on people with diagnosed OCD and articles that describe people who display OCD-related (self-reported) symptoms in order to obtain a nuanced understanding of the putative effects of pandemics.

Previous research has indicated that OCD may result in significant impaired psychosocial and occupational functionality and reduced quality of life (29). Therefore, it is important to investigate whether pandemics and their associated lockdowns—as well as other restrictive interventions such as quarantines—may worsen the symptoms of OCD in people with a previous diagnosis, and/or even cause OCD in the general population. Our review has produced unique, important findings that contribute to medical knowledge about OCD, and the results of our study have the potential to inform public-health policies that impact the lives of people with OCD.

## Methods

**Figure 1:**
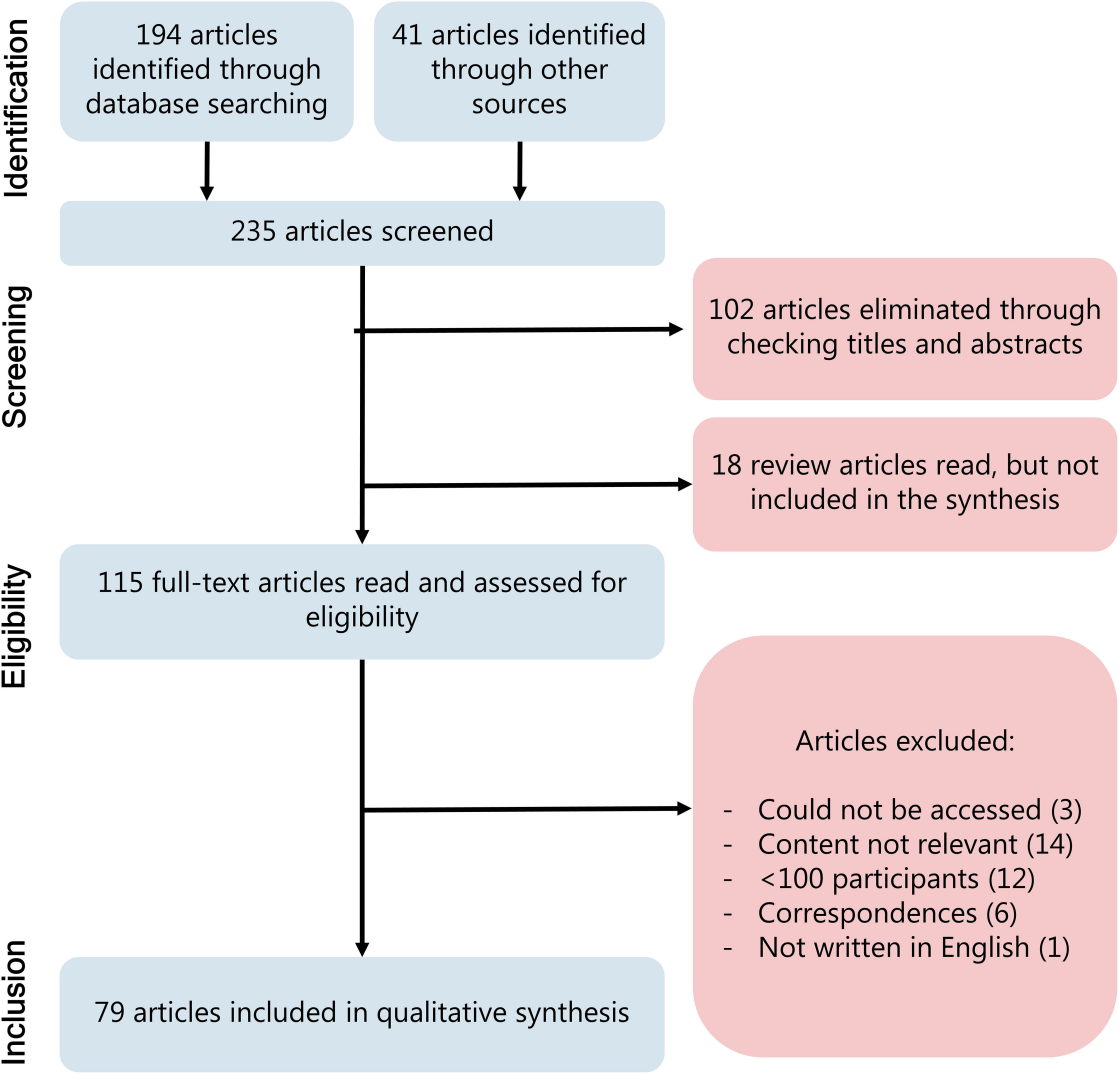
PRISMA flow diagram for study selection.

This systematic review follows the PRISMA (Preferred Reporting Items for Systematic Reviews and Meta-Analysis) guidelines (Figure 1) (30). The EndNote reference manager was used to organize references. The systematic review was not registered prior to publication.

### Eligibility Criteria

We aimed to include studies concerning the following themes, and generated our search terms accordingly:

1. Studies measuring changes in OCD-related symptoms in patients or populations during pandemics.
2. Studies investigating the mental health of patients or populations during pandemics, with a focus on OCD symptoms.

To increase the robustness of our findings, only research articles including ≥100 participants were selected (except for case reports, which were also considered to introduce a case-based qualitative aspect of the research area). Only non-review articles, published in the English language, were selected. No restrictions were set based on publication dates or study design.

### Information Sources

We conducted searches using NCBI PubMed, SCOPUS, and Google Scholar on February 9, 2021.

### Search Strategy

We searched SCOPUS with the search term: *(“Obsessive-compulsive disorder” OR “Obsessive compulsive disorder” OR ocd) AND (covid-19 OR “SARS-CoV-19” OR pandemic OR “coronavirus disease 2019”)*

We searched PubMed with the search term:

(“SARS-CoV-2”[Mesh] OR “COVID-19”[Mesh] OR “Pandemics”[Mesh] OR pandemic^*^[Text Word] OR covid^*^[Text Word] OR coronavirus [Text Word] OR “corona virus”[Text Word]) AND (“Obsessive-compulsive Disorder”[Mesh] OR “OCD”[Text Word] OR “Obsessive-compulsive disorder”[Text Word] OR “obsessive”[Text Word] OR “compulsive”[Text Word] OR “obsessive compulsive disorder”[Text Word]).

### Study Selection

After removing duplicates, the initial search resulted in 194 articles and 41 additional publications were identified through other sources, including the revision of all articles included in a recent systematic review by Guzick et al. on the topic of OCD and the COVID-19 pandemic (31). The identified publications were categorized as research articles, clinical guidelines, correspondences, case reports, and comments. We examined the abstracts of the 235 potentially eligible articles and used reference tracking for reviews to search for additional potentially eligible articles. After the exclusion of reviews and non-relevant articles based on the abstracts, 115 articles were included for full text assessment. Of these, 79 articles were included in this qualitative synthesis (Figure 1). Of the 79 articles, 59 were original research articles, which are summarized in Table 1.

**Table 1:** Original research articles related to the COVID-19 pandemic and OCD.

**References**	**Design**	**Sample size**	**Females (%)**	**Mean age**	**Country**	**Period**	**Population**	**Main findings**
**Individuals with OCD**								
Alonso et al. (32)	*Cross-sectional, case-cohort study*. Structured interviews, online, self-report survey (VAS, HDRS, DSM-5, Y-BOCS).	364	Patients: 53.5% Controls: 57.6%	Patients: 42.0 Controls: 40.8	Spain	April 27– May 25, 2020	Patients with OCD and controls from the general population	Individuals with OCD had more internalizing symptoms, suicidal thoughts, and sleep/appetite changes. OCD symptoms increased to a clinically significant degree in 40% of patients. Contamination symptoms predicted more COVID-focused symptoms and increases in OCD severity. Pre-pandemic OCD severity, depression, and social support predicted increase in OCD severity*.
Benatti et al. (33)	*Cross-sectional study*. Telephone (94%) and in-person (6%) interview.	123	44.9%	40.0	Italy	N/A (at least 3 months after the initial outbreak)	Patients with OCD	35.3% of patients experienced clinical worsening of OCD. The group with worsening OCD were characterized by the development of new obsession and/or the reoccurrence of past obsessions. The most frequent symptoms were excessive washing and cleaning in the total population.
Carmi et al. (34)	*Longitudinal study, clinical trial*. Clinical evaluation, self-report survey (CGI-I).	113	50%	33.8	Israel	April–May 2020 Reevaluation: September, 2020	Patients with OCD enrolled in a clinical trial	The majority of OCD patients with active therapy and pharmacological intervention did not report a worsening of symptoms during the COVID-19 pandemic. The majority of patients reported that COVID-19 did not impact their OCD.
Højgaard et al. (35)	*Cross-sectional study*. Self-report survey (Y-BOCS)	201	65.7%	39.7	Denmark	April 6–29, 2020	Patients with OCD	61.2% of participants reported a worsening of OCD symptoms. Being female, demonstrating contamination symptoms, and psychiatric comorbidities were associated with increased OCD severity.
Jelinek et al. (36)	*Cross-sectional study*. Online, self-report survey (PHQ-9, OCI-R)	394	73.9%	37.8	Germany	March 23–May 18, 2020	Patients with OCD	72% of the participants experienced a worsening in OCD symptoms. This deterioration was the most prominent in patients with washing compulsions. The worsening of symptoms was associated with reduced mobility and interpersonal conflicts.
Kaveladze et al. (37)	*Cross-sectional study*. Self-report survey (Dimensional Obsessive-Compulsive Scale)	196	71.4%	24.8	USA	June 28–August 10, 2020	Patients with OCD	Among a sample of adults who participated in online OCD support communities, 93% experienced symptom worsening and 96% stated having OCD made dealing with the pandemic more difficult. Rates of worsening were higher in unacceptable thought, harm, and contamination domains compared with symmetry/completeness*.
Khosravani et al. (27)	*Longitudinal study*. Online, telephone or in-person survey (DOCS, Y-BOCS, CSS)	270	57.4%	36	Iran	Before outbreak. Reevaluation: May –July, 2020	Patients with OCD	Statistically significant increase in OCD severity in all OCD dimensions during the COVID-19 pandemic compared with pre-pandemic levels. COVID-19 related stress associated with increased OCD severity.
Khosravani et al. (38)	*Cross-sectional study*. Self-report survey (CSS, PHQ-4, FCV−19S, C19P–S, SHAI, VOCI, XS, HCQ-54, OCI-R, OCS).	300	58.7%	35.8	Iran	June 1–August 15, 2020	Patients with OCD	Contamination and checking obsessive-compulsive symptoms were significantly associated with all domains of COVID-19 stress responses, including danger/contamination fears, socio-economic consequences, traumatic stress, xenophobia, and compulsive checking. Patients with OCD had significantly more COVID-related stress in all domains than patients with social anxiety and specific phobias*.
Khosravani et al. (39)	*Cross-sectional study*. Self-report survey (DOCS, Y-BOCS, CSS, PHQ-4, BSS).	304	58.6%	35.8	Iran	June 5–October 30, 2020	Patients with OCD	COVID-19-related compulsive checking and traumatic stress mediated the relationships between harm and unacceptable thought symptoms and suicidal ideation. COVID-19-related compulsive checking mediated the relationship between overall OCD severity and suicidal ideation*.
Pan et al. (40)	*Longitudinal (case-cohort) study*. Online, self-report survey (QIDS, BAI, PSWQ, DJGLS)	1,517	64%	56.1	Netherlands	Before outbreak. Reevaluation: Apr-May, 2020	Patients with OCD, anxiety or depression and controls from the general population	Individuals with OCD, anxiety and depression scored higher on the four-symptom scales compared to healthy controls from the general population both before and during the pandemic. Greater increase in symptoms was observed in healthy individuals.
Rosa-Alcázar et al. (41)	*Cross-sectional, case-control study*. Online, self-report survey (Y-BOCS, HADS, COPE-28)	237	55.7%	33.5	Spain	April 2020	Patients with OCD, and controls from the general population	Individuals with OCD reported greater use of the following: instrumental support and religion. Individuals with OCD scored higher for self-blame. Within the OCD group, presence of comorbidities was associated with denial, substance use, and self-blame. Overall, results suggest patients living with OCD could benefit from adaptive coping strategies during COVID*.
Sharma et al. (42)	*Longitudinal study*. Telephone interview (Y-BOCS, MINI, CGI-S, CTS, DSM-5, WSAS)	447	Patients with OCD before the pandemic: 35% Patients with OCD before the pandemic:37%	Patients with OCD before the pandemic: 33.0 Patients with OCD before the pandemic:32.3	India	April 26–May 12, 2020	Patients with OCD before and during the pandemic	No influence of the pandemic was observed on OCD symptoms when comparing patients with OCD during the pandemic with an independent sample of OCD patients before the pandemic. Remission rates among those with OCD were similar before and during the COVID-19 pandemic.
Storch et al. (43)	*Cross-sectional study*. Online survey filled by clinicians about their patients. (NIMH-GOCS, Y-BOCS)	232	51%	28.5	USA	July 19–August 2, 2020	Patients with OCD (data reported by their clinicians)	According to clinicians treating OCD patients with ERP before and during the pandemic, 38% of the patients had worsened symptoms, 47% stayed the same, and 10% had improved symptoms. The pandemic likely attenuated the efficacy of ERP therapy.
Toh et al. (44)	*Longitudinal case-control study*. Online, self-report survey (DASS-21, EUROHIS-QoL, OCI-R)	264	89.4%	32.9	Australia	Baseline: April 2020 Follow-up: May 2020	Patients with OCD and controls from the general population	The OCD group reported increased rates of severe depression, anxiety, reduced quality of life, and stress compared to control group between April and May 2020. Obsessive washing and checking did not increase between the two timepoints.
Tundo et al. (45)	*Cross-sectional study*. Self-report survey (SCID-5, HDRS, Y-MANIA-RS, Y-BOCS, PAAAS, BSPS)	386	59.3%	52.0	Italy	March 10–June 30, 2020	Patients with OCD, and patients suffering from other mental illness	Patients living with OCD, compared to other patients with depression, had a greater worsening of symptoms as a result of the pandemic. Differences were not found compared to other disorders*.
Wheaton et al. (46)	*Cross-sectional, case-control study*. Self-report survey (CTS, DOCS, DASS-21)	548	Patients: 79.2% Controls: 41.5%	Patients: 32.2 Controls: 38.2	USA	April 1–August 12, 2020	Patients with OCD and controls from the general population	76.2% of patients reported worsening of symptoms, and 58.3% reported COVID-19 becoming a point of their obsession. Concerns about COVID-19 were associated with OCD severity. 59.1% of patients reported COVID-19 interfering with their treatment.
**General population samples**								
Abba-Aji et al. (47)	*Cross-sectional study*. Online, self-report survey (BOCS, PSS, GAD-7, PHQ-9).	6,041	86.6%	42	Canada	March 23–30, 2020	General population	60.3% developed OCD symptoms during COVID-19 (fear of germs and viruses). Hand-washing compulsions developed in 53.8% of the population. OCD symptoms were associated with moderate/high stress, generalized anxiety disorder, and major depressive disorder.
Albertella et al. (48)	*Cross-sectional study*. Online, self-report survey (mYFAS2.0, IAT, PPCS-6, PGSI, AUDIT, OCI-R).	878	53%	32.0	Australia	May–June, 2020	General population	Younger age, greater COVID-19-related disruptions, greater psychological distress, and greater pre-COVID OCD were associated with obsessive-compulsive symptom severity*.
AlHusseini et al. (49)	*Cross-sectional study*. Online, self-report survey (PHQ-9, OCI-R)	2,187	60.5%	N/A (50% aged <35)	Saudi Arabia	N/A (during lockdown)	General population	62.4% of the respondents are likely to have OCD based on the OCI-R questionnaire. Older age, being male, being married, and having higher income were associated with increased OCD symptoms.
Cox et al. (50)	*Longitudinal study*. Online, self-report survey. (DASS, ISI, OCI-R)	369	89.1%	47.0	USA	Baseline: 2016 Follow-up: April 1–8, 2020	General public	Increase in washing and hoarding symptoms during COVID-19 pandemic compared to 2016 levels. Other OCD symptoms like ordering, neutralizing, and obsession symptoms did not change. Pre-COVID-19 insomnia was associated with an increased COVID-19 incidence of OCD symptoms.
Damirchi et al. (51)	*Cross-sectional study*. Self-report survey (STS, TDAS, MOCI, Folkman and Lazarus Coping Strategies Inventory)	300	N/A (~72–79%)	N/A (range 18–54 years)	Iran	January 21–March 19, 2020	General public	Positive correlations were found between self-talk and problem-centered coping. Inverse relationships between self-talk and emotional coping, death anxiety, and OCD symptoms were also found*.
De Pietri et al. (52)	*Cross-sectional study*. Self-report survey (HAQ, SAI, CES-D, OCI, BAI)	660	86.2%	31.1	Italy	March 26–April 9, 2020	General public	Retrospectively rated pre-pandemic obsessing and hoarding factors of the Obsessive-Compulsive Index predicted increased anxiety during the quarantine period*.
El Othman et al. (53)	*Cross-sectional study*. Self-report survey (PHQ-9, PSS-4, LAS, Y-BOCS)	386	75.9%	31.3	Lebanon	March 29–April 6, 2020	General public	Higher Y-BOCS compulsion scores were associated with more adherence to recommended hygienic practices, and higher Y-BOCS obsession scores were associated with information avoidance*.
Fontenelle et al. (54)	*Cross-sectional study*. Online, self-report survey (COROTRAS, DOCS, VOCI-MC, AAI, HRS-SR, MGHHS, SPS-R, DASS-21, WHODAS 2.0, Q-LES-Q-SF, CHIT, BIS)	829	52.6%	38.5	USA	July 29–30, 2020	General public	Statistically significant increase in OCD and related disorders, including body dysmorphic disorder and hoarding disorder compared to before pandemic levels. Based on the DOCS scale, 38.6% of respondents demonstrate severe symptoms of OCD during COVID-19, compared to 15.3% before the pandemic.
Karagöz et al. (55)	*Longitudinal study*. Interviews, self-report survey (BDI, BAI, PI-WSUR)	139	31.7%	55	Turkey	March 20–June 20, 2020	Patients with ST-Elevation Myocardial Infactrion (STEMI)	Higher contamination-related OCD was associated with delays of 120+ minutes going to the hospital for acute ST-Elevation Myocardial Infarction. Statistically significantly higher OCD subscale scores observed in March-April compared to April-June*.
Loosen et al. (56)	*Longitudinal study*. Online, self-report survey (PI-WSUR, HADS)	406	57.3%	34	United Kingdom	Baseline: April 24–May 7, 2020 Follow-up: July 15–August 15, 2020	General public	Contamination OCD symptoms in the general population appeared at similar levels as in previously reported clinical samples. Obsessive-compulsive symptoms increased across the timepoints. Information-seeking predicted increased OCD symptoms*.
Mansfield et al. (57)	*Medical record review (longitudinal design)*. Electronic health records (Clinical Research Practice Datalink Aurum).	13% of UK population (~10 M/year)	50%	N/A (aged >11)	United Kingdom	Jan 1, 2017–July 18, 2020	General public	There were statistically significantly fewer visits for OCD (and all other mental-health conditions) in July 2020, compared with January 2017*.
Mazza et al. (58)	*Cross-sectional study*. Clinical interview, self-report survey (IES-R, PCL-5, ZSDS, STAI-Y, MOS-SS, WHIIRS, OCI)	402	34.1%	57.8	Italy	April 6–June 9, 2020	COVID-19 survivors from the general public	20% of COVID-19 survivors reported symptoms of OCD. Duration of hospitalization inversely correlated with the OCI-R*.
Moreira et al. (59)	*Cross-sectional study*. Online, self-report survey (DASS-21, OCI-R)	1,280	79.8	37.1	Portugal	March 23–31, 2020	General public	Elevated self-reported OCD was reported in 12% of the sample using the OCI-R. Younger age and education were predictors of obsessive compulsive symptoms. Presence of housemates, pets, or continuing work were not*.
Mrklas et al. (60)	*Cross-sectional study*. Online, self-report survey (PSS, GAD-7, PHQ-9, BOCS)	8,267	86.2%	N/A (>90% aged >26)	Canada	March 23–May 4, 2020	General public	Self-reported prevalence rates of moderate or high stress, anxiety, and depression were 85.6, 47.0, and 44.0%, respectively. Non-healthcare workers reported higher rates of OCD symptoms compared to healthcare workers.
Munk et al. (61)	Cross-sectional study. Online, self-report survey. (BCI, BDI, SHAI, PHQ, OCI-R, WHO-5, COPE, BRS)	949	79.5%	28.9	Germany	March 27–April 3, 2020	General public	Prevalence of at least one mental-health disorder in the sample was 50.6%. 21.4% of the surveyed population reported OCD symptoms.
Ojalehto et al. (62)	*Cross-sectional study*. Online, self-report survey (CAS, DASS-21, ASI3, DOCS, CSS, BVS).	438	75.3%	30.3	USA	August 27–November 5, 2020	General public	Contamination-related OCD symptoms (DOCS contamination subscale) are statistically significant univariate predictors of COVID-19-related severe anxiety.
Quittkat et al. (63)	*Cross-sectional study*. Online, self-report survey (BDSI, DASS-D, EDE-Q, PHQ, PSWQ-d, SIAS, SPS, WI, Y-BOCS)	2,233	80.7	33.2	Germany	April 2–May 6, 2020	General public	2.1% of the population self-identified as suffering from OCD. No statistically significant changes in the level of OCD symptoms were found from November 2019 during COVID-19 (rated retrospectively). 36% of those with OCD reported worsening mental health*.
Robillard et al. (64)	*Cross-sectional study*. Online, self-report survey (PSS, DOCS, BRCS)	6,040	70.3%	51.8	Canada	April 3–May 15, 2020	General public	Obsessive-compulsive symptoms related to germs and contamination were significantly associated with increased stress levels during the outbreak*.
Samuels et al. (65)	*Cross-sectional study*. Online, self-report survey (Coronavirus Impact Scale, DY-BOCS, SMSPA, OCI-R, PHQ-4)	2,117	54%	46	USA	September 17–30, 2020	General public	COVID-19-related preventive behaviors were associated with contamination obsessions and phobias and an increase in OCD symptoms. 22.2% of responders reported high levels of contamination obsessions and 20.3% reported high levels of contamination phobias.
Wheaton et al. (17)	*Cross-sectional study*. Online, self-report survey (IUS-12, DOCS, SHAI, CTS)	720	50.3%	36.9	USA	March 2–11, 2020	General public.	Positive correlation between OCD symptoms, intolerance of uncertainty, health anxiety, and concerns about COVID-19. DOCS is a statistically significant univariate predictor of intolerance of uncertainty.
Zheng et al. (66)	*Cross-sectional study*. Online, self-report survey (Y-BOCS, SSRS, PSQI)	541	57.5%	N/A (>85% aged <45)	China	July 9–19, 2020	General public	Prevalence of demonstrating OCD symptoms was 18%. 89% of OCD patients had both obsessions and compulsions. Being unmarried, being a student, having a family history of OCD and other mental-health disorders, presence of psychiatric comorbidities, and sleep latency were risk factors for OCD.
**Pregnant women**								
Xie et al. (67)	*Cross-sectional case-control study*. Self-report survey (SCL90-R, PSQI, FES).	3,346	100%	Before pandemic cohort: 28.9 During pandemic cohort: 29.0	China	Before pandemic cohort: March 1–December 31, 2019 During pandemic cohort: January 1–August 31, 2020	Pregnant women before the pandemic, and pregnant women during the pandemic	Conflict with family was positively associated with OCD symptoms. No increases in OCD severity were noted among women who were pregnant before vs. during the pandemic*.
Yassa et al. (19)	*Longitudinal case-control study*. Self-report survey (STAI, MOCI)	304	100%	27.5	Turkey	April, 2020	Pregnant and non-pregnant women	Increased prevalence of OCD (based on high MOCI scores) in 60% of the pregnant women and in 30% of the non-pregnant women during the COVID-19 pandemic. Non-pregnant women demonstrated higher levels of anxiety during the pandemic.
**Healthcare workers**								
Ahmed et al. (68)	*Cross-sectional study*. Online, self-report survey (BAI, Y-BOCS, BDI-2)	524	57.4%	N/A (>50% aged 31–40 years)	Egypt	May 1–June 1, 2020	Healthcare workers and non-healthcare workers	7% of healthcare workers self-reported moderate to severe OCD, whereas 3% of non-healthcare workers reported moderate-to-severe OCD. OCD severity was associated with female sex, urban residency, and chronic-disease history*.
Cai et al. (69)	*Cross-sectional study*. Online, self-report survey (SCL-30, Y-BOCS, SCSQ).	616	63.8%	N/A (~90% aged 19–39 years)	China	February 5–25, 2020	Healthcare workers and non-healthcare workers	Non-healthcare workers reported statistically significantly more compulsions than healthcare workers*.
Ergenc et al. (70)	*Cross-sectional study*. Self-report survey (Obsessive-Compulsive Disorders Scale)	198	72%	COVID-group: 35.6 Non-COVID: 33.7	Turkey	N/A	Healthcare workers	Healthcare workers in the COVID-19-section scored higher on OCD, depression, and anxiety scales compared to healthcare workers in other sections.
Juan et al. (71)	*Cross-sectional study*. Online, self-report survey (IES-R, GAD-7, PHQ-9, Y-BOCS, PHQ-15)	456	70.6%	30.7	China	February 1–14, 2020.	Healthcare workers	37.5% of hospital staff experienced symptoms of OCD. Women, those with lower income, and those working on isolation wards had higher rates and more severe OCD symptoms.
Zhang et al. (72)	*Cross-sectional study*. Online, self-report survey (ISI, SCL-90-R, PHQ-4, PHQ-2, GAD-2)	2,182	64.2%	N/A (96.3% aged 18–60)	China	February 19–March 6, 2020	Healthcare workers	Medical health workers had a higher prevalence of insomnia, anxiety, depression, somatization, and OCD symptoms compared to non-medical health workers. Living in rural areas, being at risk of contact with COVID-19 patients, and having organic diseases were risk factors for OCD symptoms.
Zheng et al. (73)	*Cross-sectional study*. Online, self-report survey (PSQI, SCL-90)	207	84.5%	N/A (>60% aged >30)	China	March 1–15, 2020	Healthcare workers	25.6% of the responding medical workers reported elevated OCD symptoms*.
**Students and young adults**								
Abuhmaidan et al. (74)	*Cross-sectional study*. Online, self-report survey (SCL-90-R)	258	76.4%	N/A (91% >20 years)	United Arab Emirates	March, 2020	University students (humanities and science)	The population was characterized by low levels of mental illness. Compared to the other mental health-related dimensions (e.g., depression, anxiety), OCD symptoms were the most severe. Female students and those younger than 20 showed the poorest mental health.
Bahçecioglu et al. (75)	*Cross-sectional study*. Online, self-report survey (OCS, WCI)	628	76.4%	21	Turkey	October 4–17, 2020	University students (nursing)	Nursing students had low levels of obsession with COVID-19, and demonstrated moderate coping skills. On average, female students were more stressed than male students.
Chen et al. (76)	*Cross-sectional study*. Online, self-report survey (CCMD-3, Brief Response Questionnaire)	992	52.8%	19.3	China	March 27, 2020	University students	From a population of young people living in isolation for two months, 6% were categorized as high-risk, 63% were medium-risk, and 31% were low-risk of developing a mental illness. Unhealthy behaviors (e.g., smoking, alcohol consumption) increased the risk for psychological problems. Negative pandemic information increased anxiety, controllability, and vulnerability.
Darvishi et al. (77)	*Cross-sectional study*. Self-report survey (MOCI, CEQ)	150	64.7%	16.7	Iran	N/A (before July 2020)	High-school and pre-university students	67% of subjects may have demonstrated OCD symptoms. Prevalence in women is higher than in men (72.1 vs. 60.3%). Washing compulsion is the most common symptom.
Ji et al. (78)	*Longitudinal study*. Online, self-report survey (Y-BOCS, SAS)	13,478	65.4%	21.3	China	Survey 1: February 8, 2020 Survey 2: March 15, 2020 Survey 3: April 30, 2020	University students (medical and non-medical)	Higher prevalence of OCD and anxiety levels in March (11.3%) compared to April (3.6%) and May (3.5%). Male students had higher prevalence of OCD symptoms compared to female students at all timepoints.
Jiang (79)	*Cross-sectional study*. Online, self-report survey (SCL-90)	Participants: 472 Population norm: 12,160	51.9%	N/A (aged 17–22 years)	China	February 10, 2020	University students	Students had increased levels of obsessive behaviors compared with the general population. Students had insufficient knowledge about COVID-19 and demonstrate high-risk perceptions (i.e., high levels of fear of the virus and getting infected).
Knowles et al. (80)	*Longitudinal study*. Self-report survey (PI, OCI-R, CAI, CSBS, IAI, ISBS)	108	75%	19.6	USA	Baseline: January 2020 Follow-up: February 27–March 26, 2020	University students	COVID-19 anxiety and precautionary behaviors were higher than for influenza. Mean levels of OCD washing symptoms increased between January 2020 and March 2020.
Meda et al. (81)	*Longitudinal study*. Self-report survey (BDI-2, BAI, OCI-R, EHQ, EDI-3).	358	79.9%	21.3	Italy	Baseline: October–December, 2019 Follow-up: April–June, 2020	University students	Scores on the OCI-R were reduced over the course of the pandemic, independent of history of mental-health disorder or the participant's sex. 86% of the students did not experience a worsening of symptoms.*
Wheaton et al. (82)	*Cross-sectional study*. Online, self-report survey (ECS, CTS, DASS-21, OCI-R)	603	87.6%	22.9	USA	April 5–May 13, 2020	University students	Greater susceptibility to emotion contagion was associated with concerns about COVID-19, depression, anxiety, stress, and OCD symptoms. Emotion contagion moderated relationship between COVID-19-related media consumption and OCD symptoms.*
**Children and adolescents**								
Cho et al. (83)	*Longitudinal study*. Self-report survey (SHAPS, DTS, CASI, UPPS Impulsive Behavioral Scale, RCADS)	2,120	61.2%	21.2 (at follow-up)	USA	Baseline: 2016 Follow-up: May–August, 2020	Adolescents	High school students completed substance use assessments in 2016 and again in May-August 2020. Substance use in adolescence did not predict OCD severity in young adulthood during the pandemic.*
McKune et al. (84)	*Cross-sectional study*. Self-report survey.	280	51.8%	N/A (range 5-18)	USA	April 2020	School-age children	32.1% of the population were at risk and 8.9% at high risk of OCD. OCD symptoms were associated with loss of household income, female sex, and younger age.*
Nissen et al. (85)	*Cross-sectional study*. Patient records, self-report survey (Y-BOCS)	102	Clinical group (CG): 63.1% Survey group (SG): 66.7%	Clinical group (CG): 14.9 Survey group (SG): 14.1	Denmark	April–May 2020	Children newly diagnosed with OCD (CG), and children diagnosed with OCD years ago (SG)	Children newly diagnosed or long-term diagnosed with OCD both experienced worsening of OCD, anxiety, depression, and avoidance behavior. Changes in the total OCD severity scores correlated with worsening levels of anxiety and depression. These findings were the most pronounced in children with early onset of ADHD and family history of ADHD.
Seçer et al. (86)	*Cross-sectional study*. Online, self-report survey (OCI-CV, ERS, Depression and Anxiety Scale for Children, Fear of COVID-19 Scale)	598	61.1%	16.4	Turkey	N/A	Adolescents	Increased OCD symptoms in adolescents. Fear of COVID-19 is associated with the development of OCD symptoms and is a predictor of depression- and anxiety-related symptoms. Experiential avoidance mediates the relationship between fear of COVID-19 and OCD symptoms.
**Previous pandemics and OCD**								
Brand et al. (87)	*Cross-sectional study*. Self-report survey (OCI-R, ASI3, Swine Flu inventory, OBQ-44, DS-R)	393	68%	20.1	USA	November 2009–March 2011	University students	OCD symptoms predicted Swine Flu-related fears. Disgust sensitivity mediated the relationship between both OCD beliefs and OCD symptoms and Swine Flu-related fears.

*Summary extracted or adapted from the systematic review: A.G. Guzick, A. Candelari, A.D. Wiese, S.C. Schneider, W.K. Goodman, and E.A. Storch, Obsessive–Compulsive Disorder During the COVID-19 Pandemic: a Systematic Review. Current psychiatry reports 23 (2021) 1-10.

*AAI, Appearance Anxiety Inventory; ASI3, Anxiety Sensitivity Index-3; AUDIT, Alcohol Use Disorders Identification Test; BAI, Beck Anxiety Inventory; BCI, Behavioral Item Regarding Corona; BDI, Beck-Depression-Inventory; BDSI, Body Dysmorphic Symptoms Inventory; BIS, Barratt Impulsivity Scale; BOCS, Brief Obsessive-Compulsive Scale; BRCS, Brief Resilient Coping Scale; BRS, Brief Resilience Scale; BSPS, Brief Social Phobia Scale; BSS, Beck Scale for Suicidal Ideation; BVS, Body Vigilance Scale; C19P–S, COVID-19 Phobia Scale; CAHSA, Continuum of Auditory Hallucinations – State Assessment; CAI, Coronavirus Anxiety Inventory; CAS, Coronavirus Anxiety Scale; CASI, Childhood Anxiety Sensitivity Index; CCMD-3, Chinese Classification of Mental Disorders; CEQ, Cognitive Errors Questionnaire; CES-D, Center for Epidemiologic Studies Depression Scale; CGI, Clinical Global Impressions; CGI-I, Global Clinical Impression–Improvement; CGI-S, Clinical Global Impression–Severity; CHIT, Cambridge-Chicago Compulsivity Trait Scale; COPE, Coping Survey; COROTRAS, Coronavirus Traumatic and Stressful Life Events Scale; CSBS, Coronavirus Safety Behaviors Scale; CSS, Contamination Cognitions Scale; CSS, COVID Stress Scale; CTS, COVID-19 Threat Scale; DASS, Depression Anxiety Stress Scales; DASS-D, Depression Anxiety Stress Scales – Depression Subscale; DJGLS, De Jong Gierveld Loneliness Scale; DOCS, Dimensional Obsessive Compulsive Scale; DSM-5, The Diagnostic and Statistical Manual of Mental Disorders; DS-R, Disgust Scale-Revised; DTS, Distress Tolerance Scale; DY-BOCS, Dimensional Yale-Brown Obsessive-Compulsive Scale; ECS, Emotion Contagion Scale; EDE-Q, Eating Disorder Examination-Questionnaire – 2nd Edition; EDI-3, Eating Disorder Inventory – 3; EHQ, Eating Habits Questionnaire; ERS, Emotion Reactivity Scale; EUROHIS-QoL, European Health Interview Surveys-Quality of Life; FCV−19S, Fear of COVID-19 Scale; FES, Family Environment Scale; GAD-7/GAD-2, Generalized Anxiety Disorder Assessment; HADS, Hospital Anxiety and Depression Scale; HAQ, Health Anxiety questionnaire; HCQ-54, Health Concerns Questionnaire-54; HDRS, Hamilton Depression Rating Scale; HRS-SR, Hoarding Rating Scale-Self Report; IAI, Influenza Anxiety Inventory; IAT, Young's Internet Addiction Test; IES-R, Impact of Events Scale-Revised; ISBS, Influenza Safety Behavior Scale; ISI, Insomnia Severity Index; IUS-12, Intolerance of Uncertainty Scale; LAS, Lebanese Anxiety Scale; MGHHS, Massachusetts General Hospital Hairpulling Scale; MINI, Mini International Neuropsychiatric Interview; MOCI, Maudsley Obsessive-Compulsive Inventory; MOS-SS, Medical Outcomes Study Sleep Scale; mYFAS2.0, Modified Yale Food Addiction Scale 2.0; N/A, Not available information; NIMH-GOCS, National Institute of Mental Health Global Obsessive Compulsive Scale; OBQ-44, Obsessional Beliefs Questionnaire-44; OCI-CV, Obsessive Compulsive Inventory – Child Version; OCI-CV, Obsessive Compulsive Inventory–Child Version; OCI-R, Obsessive-Compulsive Inventory-Revised; OCS, Obsession with COVID-19 Scale; PAAAS, Panic Attack and Anticipatory Anxiety Scale; PCL-5, PTSD Checklist for DSM-5; PGSI, Problem Gambling Severity Index; PHQ-2/PHQ-4/PHQ-9/PHQ-15, Patient Health Questionnaire; PI, Padua Inventory; PI-WSUR, Padua Inventory-Washington State University Revision; PPCS-6, Short Version of the Problematic Pornography Consumption Scale; PSQI, Pittsburgh Sleep Quality Index; PSS, Perceived Stress Scale; PSWQ/ PSWQ-d, Penn State Worry Questionnaire; QIDS, Quick Inventory of Depressive Symptoms; Q-LES-Q-SF, Quality of Life, Enjoyment, and Satisfaction Questionnaire-Short Form; RCADS, Revised Children's Anxiety and Depression Scales; SAI, Social Anxiety Inventory; SAS, Zung Self-Rating Anxiety Scale; SCID-5, Structured Clinical Interview for DSM-5; SCL-30, Symptom Check List-30; SCL90-R, Symptom Checklist-90 Revised; SCSQ, Simplified Coping Style Questionnaire; SHAI, Short Health Anxiety Inventory; SHAPS, Snaith Hamilton Pleasure Capacity Scale; SIAS, Social Interaction Anxiety Scale; SMSPA, Severity Measure for Specific Phobia–Adult; SPS, Social Phobia Scale; SPS-R, Skin Picking Scale-Revised; SSRS, Social Support Rating Scale; STAI/ STAI-Y, The State-Trait Anxiety Inventory; STS, Self-Talk Scale; TDAS, Templer Death Anxiety Scale; UPPS, UPPS Impulsive Behavioral Scale; VAS, Visual Analog Scale; VOCI-MC, Vancouver Obsessional Compulsive Inventory – Mental Contamination; VOCI-MC, Vancouver Obsessional Compulsive Inventory; WCI, Ways of Coping Inventory; WHIIRS, Women's Health Initiative Insomnia Rating Scale; WHO-5, Well-being Index; WHODAS 2.0, World Health Organization Disability Assessment Schedule 2.0; WI, Whitely Index; WSAS, Work and Social Adjustment Scale; XS, xenophobia scale; Y-BOCS, Yale-Brown Obsessive-Compulsive Scale; Y-MANIA, Y-MANIA Rating Scales; Y-MANIA-RS, Y-MANIA Rating Scales; ZSDS, Zung Self-Rating Depression Scale*.

### Data Collection

The screening of titles and abstracts was conducted by two co-authors (ESL and TVV) independently, and conflicts in this screening process were resolved by including any articles co-authors selected for full-text assessment. Quality assessment was undertaken by TVV.

### Data Extraction

We extracted the following data from the full-text clinical research articles: study design, method of exposure and outcome ascertainment, demographic characteristics (mean age and percent female), sample size, country, period of data collection, and main findings. The Newcastle-Ottawa Scale was used to assess the overall quality of the included clinical research articles based on nine aspects related to study selection, comparability, and outcome assessment (88).

## Results and Discussion

We analyzed a total of 79 articles on OCD and pandemics. From these, 59 were research articles (Table 1), six were case reports, and 14 articles were communications or clinical guidelines. Of the 59 research articles, 16 examined individuals diagnosed with OCD prior to the pandemic. Twenty-one articles examined the general population of a specific country. Two articles investigated how pregnant women are affected by the pandemic, six studied healthcare workers, nine focused on students and young adults, and four articles investigated COVID-19 in children and adolescents. Only one study was identified on OCD during previous pandemics. Six articles were case reports of individuals with OCD during COVID-19. An additional 12 articles were letters, editorials, and comments with relevant discussion points, and two articles were clinical guidelines on how medical consultations and treatments were being modified during the COVID-19 pandemic. The quality assessment of the 59 original research articles is presented in Supplementary Table 1.

Overall, the 59 research articles on various demographic groups indicated that the populations studied experienced a worsening of their OCD symptoms as well as increased symptoms of other mental-health disorders and a reduced quality of life. The six case reports provided examples of how the clinical impairments might look among individuals with OCD. The 12 letters reported a more varied picture, arguing that some individuals may be experiencing worsening symptoms during the pandemic, while others were not significantly affected; some may even experience improved mental health. The two clinical guidelines provided information on how to engage with and treat individuals with OCD during this period. We used the data collected from the various articles to answer our three main questions presented in the introduction.

### The Prevalence of OCD and Its Symptoms Before and During the COVID-19 Pandemic

The estimated lifetime prevalence of OCD is around 2–3% globally (66). According to a study from 2003, the estimated prevalence of OCD was 1.2% among the adult U.S. population (89). We were unable to identify comprehensive studies on the prevalence of OCD globally or nationwide since the pandemic started. Only the prevalence of OCD (or its symptoms) in specific demographic groups and specific nationalities has been investigated so far and, as it is discussed in the following section, research shows a tendency towards increased OCD symptoms in all investigated demographic groups.

In 2020, Zheng et al. (66) investigated the prevalence of OCD symptoms in Wuhan, China. In July, three months after reopening after lockdown, 17.93% of the investigated population had symptoms of OCD, but unfortunately there was no pre-pandemic statistic for comparison. While this figure is certainly higher compared to the estimated 1.2% OCD prevalence in the U.S. population, a significantly larger percentage of populations (14–29%) has been shown to demonstrate mild symptoms of OCD even prior to the pandemic (24). The study found that being single, student, having comorbid mental disorders, family history of OCD, and sleep latency were all associated with OCD.

In Iran, Khosravani et al. (27) found increased levels of OCD severity when comparing pre-pandemic and pandemic levels in patients diagnosed with OCD prior to the pandemic. The results of the study indicated that the increased severity of OCD symptoms was primarily was primarily due to stress induced by the current pandemic.

A study by Munk et al. (61) found a higher prevalence of OCD symptoms in Germany during the first weeks of the pandemic (March, 2020) compared to the reported prevalence pre-pandemic; 21.4% of the participants expressed clinically-significant OCD symptoms during the pandemic compared to 3.6% reported in the general population. Prevalence of depression and general anxiety disorder were also significantly higher than what was reported in the general population, which again indicates an overall initial stress response to the pandemic.

In India, Sharma et al. investigated relapse rates in individuals diagnosed with OCD prior to the pandemic compared to a control group (42). The authors did not find worsening in severity of illness nor did they find increased relapse rates. Also, very few patients developed COVID-19-related OCD symptoms. They argue that this might be because data collection was conducted relatively early in the pandemic (April-May, 2020), that patients were already on medication, and/or that the lockdown and various restrictions and recommendations might have limited their exposure to COVID-19 (42).

With regards to the etiology of OCD during COVID-19, many articles in our review report various risk factors that triggered OCD symptoms during the current pandemic (16, 71, 74, 75, 77). Banerjee (16) lists seven factors that may play a role in the worsening of OCD symptoms: 1. an increased demand for hand-washing; 2. recommended hand-washing steps that may reinforce ritualistic patterns; 3. recommended hand-washing after suspected exposures, which may provide cognitive justification; 4. the prompting of family to ensure strict hygiene measures; 5. the media's regular reporting of possible sources of contamination; 6. increased ruminations and repeated washing, which can become normalized during the pandemic; and 7. stocking protective equipment and disinfectants, which may increase hoarding symptoms (16).

An interesting point by Banerjee is that in previous pandemics like Severe Acute Respiratory Syndrome (SARS), Middle Eastern Respiratory Syndrome (MERS), and influenza, the worsening of OCD symptoms has advanced up to 6–12 months *after* the end of the outbreak. They argue that symptoms might not be evident during a pandemic due to under-detection and alternate public-health priorities. As several studies in our review suggested, some patients with OCD may not seek treatment and follow-up meetings because of fear of contamination, stigma, or lack of knowledge about what is excessive cleaning/washing; this is a possible explanation for an increase in their symptoms (27, 90). The largest study identified in our review by Mansfield et al. (57), which investigated electronic health records of millions of individuals from the United Kingdom before and during the pandemic, also observed fewer visits related to OCD during the pandemic compared to the years before. Thus, it is important that clinicians follow up with patients who have been previously diagnosed with OCD but who are not in active treatment. As the COVID-19 pandemic is still active, it is quite possible that we will see an increase in OCD incidence once the pandemic has ceased.

### Worsening of OCD in Specific Demographic Groups and Personal Characteristics

Two letters (10, 91) report that responses from patients with OCD have been varied; some people experience increased anxiety while others feel validated in their concerns and/or reassured by the strict guidelines (10). Based on such findings, Perkes et al. argue that the recommended measures may be more stressful to those without OCD compared to individuals who are already accustomed to these practices (91).

In the 16 research articles on the effects of COVID-19 on *patients diagnosed with OCD prior to the pandemic*, the clinical landscape has been more homogenous; most of these articles found a clinically significant increase in OCD symptomatology in patients suffering from OCD (27, 32, 33, 35–38, 45, 46). However, some articles did not, or reported mixed results (34, 40, 42–44). The findings from these studies suggest that the COVID-19 pandemic represents a stressor for many individuals with OCD resulting in increased OCD symptoms, although not all of the studies identified in our search fully support this notion. For example, in some patients with clinical worsening of OCD symptoms, their symptoms were only a part of a larger clinical impairment (33). Of all the previously characterized subgroups of individuals with OCD, *those with washing and cleaning compulsions* have had the most severe impairment during the current pandemic (33, 36, 47, 77, 79, 80). Research indicates that COVID-19-related stress was also associated with increased OCD severity (27) and that, compared to the general population, individuals with OCD were more likely to have moderate/high stress, general anxiety disorder, and depression (47, 78).

When investigating OCD symptoms among the general population, some studies (17, 50, 61, 66) found a small increase in OCD symptoms after the pandemic's initial outbreak, with hand-washing symptoms and contamination obsessions being predominant. The results of these studies indicate that many aspects of OCD remain unaffected by the COVID-19 pandemic, at least among the general population. However, there may also be an increased prevalence of other mental-health disorders; this indicates that not only are certain at-risk groups under psychological distress but also that the pandemic is affecting all groups of society (61, 66). This is supported by the majority of studies of students and young adults, which generally showed a complex influence of COVID-19 on mental health. Although not all of these studies indicated an increase in OCD symptoms, they generally indicated that the young adults' mental health did in fact decline (74–80).

Several studies found that *pregnant women and medical workers* are more susceptible to OCD symptoms compared to the general public (71, 72, 92). A number of studies found an increase in obsessive-compulsive symptoms and anxiety levels among pregnant women and healthcare workers (19, 70–72). These results highlight the necessity of adequate working conditions and recovery programs so that medical workers may progress toward improved psychological wellbeing as well as an increased focus on the mental health of pregnant women (70–72).

*Children and adolescents* were also identified as a risk group. Research indicates an association between negative and traumatic childhood experiences and OCD symptoms in adulthood (93). The adverse experiences during the current pandemic may have an immediate negative impact on children and adolescents both with and without OCD, especially among those with early age of onset and a family history of psychiatric disorders (85, 86). Reactions seemed to be more severe if the child did not have access to a psychiatric facility. These effects carry a high risk of long-term consequences for the individuals affected (93); hence, we believe that this at-risk group needs closer attention and further research.

Even though good hand hygiene was one of the first precautionary behaviors consistently recommended by multiple national governments, none of the studies we identified examined the physical consequences of OCD with regards to *compulsive hand-washing*. Nor did any studies examine the physical consequences of excessive *hand sanitizing*. Studies published prior to the COVID-19 pandemic have shown that compulsive hand-washing often induces severe skin damage and hand eczema (94, 95). As such, there is often a high burden in the field of dermatology due to patients with obsessive hand-washing and fear of contamination. Some of the research suggests that recommendations for good hygiene provide patients with cognitive justification, which consequently results in cases of hand eczema (16, 95). Moreover, Xerfan et al. (94) suggest an interaction between hand eczema, sleep disturbances, and OCD. Research prior to the current pandemic has also reported a link between sleep disturbances and OCD, either directly or indirectly via other mental-health disorders. These findings align with our findings from the literature during the current pandemic, in which sleep disturbances may also be associated with OCD symptoms (50, 66).

The six case reports included in our review provide examples of how individuals with OCD may react to their circumstances during the current pandemic; we chose to include these reports in our systematic review to exemplify how the pandemic might have impacted individuals living with OCD. Several of these reports (4, 96–98) reported an exacerbation of symptoms in patients diagnosed with OCD. The main symptoms were self-isolation, avoidance of certain foods, and excessive hand-washing, and cleaning. In the most severe cases (4, 96), individuals reported panic symptoms and suicidal ideation or attempts. Previous studies have suggested that, of all OCD symptoms, patients with predominant contamination obsessions and compulsive cleaning tend to exhibit the highest rates of suicidality (99). In all of the case reports, patients benefitted from a combination of pharmaceutical and psychological treatment in healthcare facilities. Nevertheless, these reports are a warning that patients with OCD should be more closely monitored to prevent severe mental-health consequences from the pandemic.

Two of the case reports described possible improvements of OCD symptoms during the pandemic; in the case report by Conrad et al. (40), five adolescent female patients diagnosed with OCD attended an experiment without a control group consisting of cognitive-behavioral group therapy for a period of 12 weeks in the U.S. With social support, education about OCD symptoms, coping, and adaptions during lockdown, these patients recovered and improved their outcomes during the therapy. In the report by Kuckertz et al. (47), eight OCD patients in a residential treatment program reported various experiences. None of them had a significant decline in their quality of life during the pandemic; in fact, most patients experienced an improvement of their symptoms.

It is worth noting that, in these small interventions, patients with close, continuing contact with healthcare providers seemed to be more resilient and more equipped to meet the challenges posed by the pandemic. In contrast, those patients who experienced an acute exacerbation of their OCD symptoms were typically those who had been diagnosed prior to the pandemic or those who did not receive regular follow-ups and support from healthcare professionals.

Only one study from a previous pandemic (the H1N1 “Swine Flu” pandemic) examined the effects of pandemics on OCD symptomatology. Brand et al. (87) found a relationship between OCD symptoms and a fear of the Swine Flu. However, the authors did not specifically evaluate the effects of the pandemic on the worsening of symptoms in individuals with OCD, nor the rates of OCD.

### The Management of OCD During Pandemics

Fontenelle et al. (100) hypothesize that cognitive behavior therapy (CBT) with Exposure and Response Prevention (ERP) may clash with the public-health recommendations regarding hygiene and protective equipment during the current pandemic, as an active element of ERP is to expose patients to feared objects. Storch et al. (43) oppose this, stating that empirical support for the abovementioned standpoint is lacking, and that there are no negative consequences of ERP during COVID-19; however, the authors acknowledge that ERP treatment needs to be adjusted to the current situation. They suggest that clinicians should continue to assess compulsions and obsessions, and that exposures should target *excessive* rituals from core obsessions, which are most often not COVID-19-related (43). Some clinicians have advised that ERP therapy should be conducted online, although the effects of such interventions are yet to be examined (10, 43, 100). In cases of a fear of COVID-19 itself, when planning treatment, clinicians will likely need to weigh the risks of contracting COVID-19 vs. the benefits of overcoming OCD (101). To educate patients on common symptoms and to prevent obsessions and compulsions, Farhan et al. (22) proposed the utilization of an innovative online chatbot. Many clinicians encourage educating both individuals with OCD and the general public in stress management (10, 61, 66).

Various other treatment strategies have been investigated and/or proposed. Chen et al. (66) proposed a six-step intervention strategy, namely: 1. Deliver positive information about the pandemic in order to reduce the abnormally increased risk perception among individuals with OCD; 2. Reduce negative behavioral responses to stress that may worsen OCD symptoms (e.g., smoking, drinking, over-eating, and taking medications); 3. Educate individuals at-risk of OCD about stress management; 4. Improve family relationships and community support; 5. Increase positive behaviors like being active, working, or studying; and 6. Adjust expectations to relieve stress. Treatment should be individually tailored; i.e., when treating individuals with OCD, some—or all—of these steps could be implemented, depending on the severity of symptoms.

**Figure 2:**
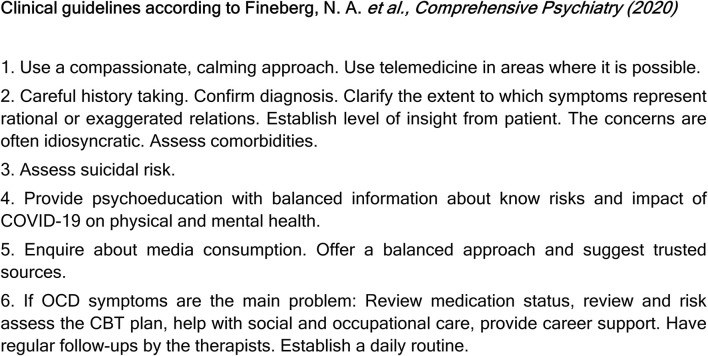
Clinical guidelines for the treatment of obsessive-compulsive disorder during the COVID-19 pandemic.

When it comes to treatment, many articles advise following the clinical guidelines proposed by Fineberg et al. (10). These guidelines were written by a working group of clinical experts based on empirical evidence, and they emphasize the importance of focusing on resilience and interventions that maintain a calm attitude, build community, and sustain hope (Figure 2). However, Farhan et al. (22) were skeptical of these guidelines, arguing that they are of little help in reality due to limited resources, high cost, and a lack of therapists in many countries worldwide. According to these authors, some of the public-health recommendations and preventive measures implemented during the pandemic have been mostly targeted at healthy people, and ambiguous terminology may worsen symptoms in individuals with OCD. They also provide an example: “*The US Centers for Disease Control and Prevention recommends washing hands for at least 20 seconds and disinfecting surfaces daily, whereas WHO suggests cleaning hands regularly and thoroughly.”* As individuals with OCD often overestimate risks, recommendations on hygiene should be precise and with limits.

Several studies in our review warn about the aftermath of the current pandemic. As there is often latency in diagnosis, consecutively worsening prognosis, and resistance to treatment, early identification and prevention of OCD symptoms is of the utmost importance (18). Pozza et al. suggest that early intervention may be especially helpful for individuals with sub-threshold OCD symptoms (18). People at-risk need to receive education about the COVID-19 virus as well as information that the public-health authorities' recommendations are *sufficient* and that excessive behaviors do not further reduce risk (102). While no current evidence suggests that there will be an increase in OCD patients after the pandemic, helping the general population to identify warning signs of OCD (e.g., in close relatives and friends) might be useful for prevention (101).

### Limitations and Knowledge Gaps

The literature on changes in OCD symptoms during the COVID-19 pandemic is still limited, and the studies included in our review have several limitations as revealed by the quality assessment (Supplementary Table 1).

First, the majority of the identified studies (76%, 45/59) are cross-sectional in nature and data were collected during the first months of the pandemic. Due to cross-sectional designs, most of the studies revealed statistical associations that are unable to demonstrate causal relationships (70, 74, 86). Of note, several studies are planning longitudinal follow-ups of their populations. Further longitudinal studies are warranted to examine the long-term effects of public-health recommendations related to the COVID-19 pandemic on OCD symptoms.

Second, apart from one article, most studies (98%, 58/59) included self-reported questionnaires as the means to assess outcomes; this data-collection method reduces accuracy and likely biases the results compared to data collection *via*, e.g., structural clinical interviews. As one study stated, it is possible that individuals with OCD were more likely to participate in some of the studies during the pandemic, potentially overestimating their own symptoms (36). Furthermore, an increase in OCD symptoms likely reflects the real threat of the SARS-CoV-2 virus, and not necessarily obsessive-compulsive trends in the populations (47).

Third, there is a lack of comprehensive and comparative assessments of incidence rates of OCD in populations before and during the COVID-19 pandemic. We were unable to identify recent statistics on the prevalence or incidence of OCD globally. Furthermore, most of the current research studies on the prevalence of OCD or its symptoms after the COVID-19 outbreak in certain demographic groups do not report pre-pandemic statistics for comparison. Hence, it is difficult to reach conclusions on the effect of the pandemic on the incidence of OCD cases in populations.

Fourth, enrollment in some of the studies (42, 80, 85) occurred over a longer period, resulting in heterogeneous study populations. While these populations allow for a wider generalizability of results, more focused studies are needed to gain a complete understanding about key aspects of OCD pathophysiology during the current pandemic. Related to this point, further studies will need to include a broad variety of demographic groups—cross-cultural and multinational—in order to gain more extensive and generalizable knowledge on general populations and OCD during the COVID-19 pandemic. Only three out of all articles analyzed representative samples or took measures to ensure representative samples of the underlying populations; conversely, most studies (95%, 56/59) did not utilize representative samples. This review has examined studies focusing on several at-risk subpopulations during the pandemic: patients with pre-existing OCD, children, adolescents, and pregnant women. However, we did not identify any articles that investigated OCD symptomatology specifically in older populations, another key high-risk group.

Last, most articles reviewed in this study investigate contamination-related OCD symptoms and were less focused on other types of OCD. Further research is warranted on less studied clinical manifestations.

As with most systematic reviews, there is a risk that relevant articles were missed. To mitigate this risk, two authors (ESL and TVV) scanned the literature and read all titles and abstracts to narrow down the search results to those articles that were read in full. During the drafting phase, another systematic review on OCD and COVID-19 was published (31); we incorporated all of the original research articles identified by these authors and their key findings in this report. We also acknowledge that scientific literature related to the COVID-19 pandemic is rapidly accumulating; thus, since conducting our final search, it is likely that additional research has been published that might nuance our findings or address the knowledge gaps we identified above.

## Conclusions

Despite increased focus on OCD during this pandemic, literature is still limited. A recently released systematic review on various aspects of OCD during the COVID-19 pandemic highlights the exacerbation of OCD-related symptoms and the emergence of new symptoms during the pandemic. Most important, it emphasizes the importance of continuing established evidence-based therapies during the pandemic (31). We add to this body of evidence by our review of the literature; current evidence from research articles suggests that both people with and without OCD prior to the pandemic show increased symptoms of OCD during the COVID-19 pandemic. High-risk groups include OCD patients in remission/recovery, geriatrics, pregnant women, children and adolescents, and healthcare professionals. Of all demographic groups included in the articles, individuals with diagnosed OCD prior to the pandemic with hand-washing and cleaning compulsions have had the most severe impairment during the pandemic.

To prevent worsening of symptoms in OCD patients, clinicians are encouraged to check in with their patients and adjust treatment based on the specific needs of the patient. As early intervention is key to prevent new cases, the articles suggest the need for sufficient education of the general population on both stress management, OCD symptoms, and on the COVID-19 pandemic.

OCD is an extremely heterogeneous and complex disorder. While not all individuals are affected negatively by the current conditions, most of our results show a worsening of OCD symptoms in the examined populations. The time frame makes any conclusion even more complex, since OCD develops and presents itself slowly. Due to the acute nature of COVID-19, and because the pandemic is still ongoing, we do not yet have long term data on the putative effects of the pandemic and its associated lockdowns. Multinational and cross-cultural, longitudinal studies are warranted to address the extensive remaining knowledge gaps.

## Data Availability Statement

The original contributions presented in the study are included in the article/Supplementary Material, further inquiries can be directed to the corresponding author/s.

## Author Contributions

EL drafted the article. EL and TV conducted the article search. TV conducted the quality assessment. TV and AC provided supervision and edited the article. All authors approved the final version of the manuscript.

## Conflict of Interest

The authors declare that the research was conducted in the absence of any commercial or financial relationships that could be construed as a potential conflict of interest.

## Publisher's Note

All claims expressed in this article are solely those of the authors and do not necessarily represent those of their affiliated organizations, or those of the publisher, the editors and the reviewers. Any product that may be evaluated in this article, or claim that may be made by its manufacturer, is not guaranteed or endorsed by the publisher.
